# Efficacy of peroral endoscopic myotomy for improving sleep problems in patients with achalasia

**DOI:** 10.1002/deo2.70064

**Published:** 2025-01-20

**Authors:** Toshihiro Ohmiya, Hironari Shiwaku, Hiroki Okada, Akio Shiwaku, Suguru Hasegawa

**Affiliations:** ^1^ Department of Gastroenterological Surgery Fukuoka University Faculty of Medicine Fukuoka Japan

**Keywords:** achalasia, gastroesophageal reflux disease, peroral endoscopic myotomy, quality of life, sleep problem

## Abstract

**Objectives:**

Achalasia is an esophageal motility disorder of unknown etiology. However, no studies have determined the populations in which sleep problems occur and whether they are improved by peroral endoscopic myotomy (POEM). We investigated the rate of sleep problems assessed by GERD‐Q (AGQ) in achalasia patients, evaluated whether POEM improves these issues, and identified factors associated with sleep improvement after POEM.

**Methods:**

We retrospectively analyzed the data of patients who were diagnosed with achalasia and who underwent POEM at a single institution between March 2016 and December 2020. We examined the Eckardt symptom score and the GERD‐Q before and 3 months after POEM to assess the presence of sleep problems (AGQ) and other symptoms. The univariate logistic regression analysis was performed to identify factors associated with sleep problem (AGQ) improvement after POEM.

**Results:**

A total of 177 patients were included. The average age was 52.6 ± 17.2 years. Preoperatively, dysphagia (172 [97.2%]), regurgitation (123 [69.5%]), sleep problems (AGQ; 110 [62.1%]), chest pain (102 [57.6%]), and weight loss (83 [46.9%]) were observed. Before POEM, 62.1% of patients experienced sleep problems (AGQ) compared with 9.6% after POEM (*p* < 0.0001). Postoperative dysphagia and regurgitation were significant factors determining whether patients continued to experience sleep problems (AGQ) after POEM.

**Conclusions:**

Sleep problems (AGQ) were the third most common symptom in > 60% of patients with achalasia. Improving dysphagia and regurgitation using the POEM procedure improved sleep problems (AGQ).

## INTRODUCTION

Achalasia is an esophageal motility disorder of unknown etiology. Achalasia is characterized by impaired lower esophageal sphincter (LES) relaxation and impaired esophageal body peristalsis.^1^, ^2^, ^3^ The main symptoms of achalasia include dysphagia, regurgitation, chest pain, and weight loss according to the Eckardt symptom score. But, in the actual clinical setting, patients complaining of sleep problems are often encountered. However, no studies have comprehensively examined the patient populations in which sleep problems caused by achalasia occur and whether they are improved by peroral endoscopic myotomy (POEM). In this study, we investigated the rate of sleep problems assessed by GERD‐Q (AGQ) in patients with achalasia, assessed whether POEM improves these sleep problems (AGQ), and further determined the factors associated with the improvement in sleep problems (AGQ) in these patients after POEM.

## METHODS

### Study design

**FIGURE 1:**
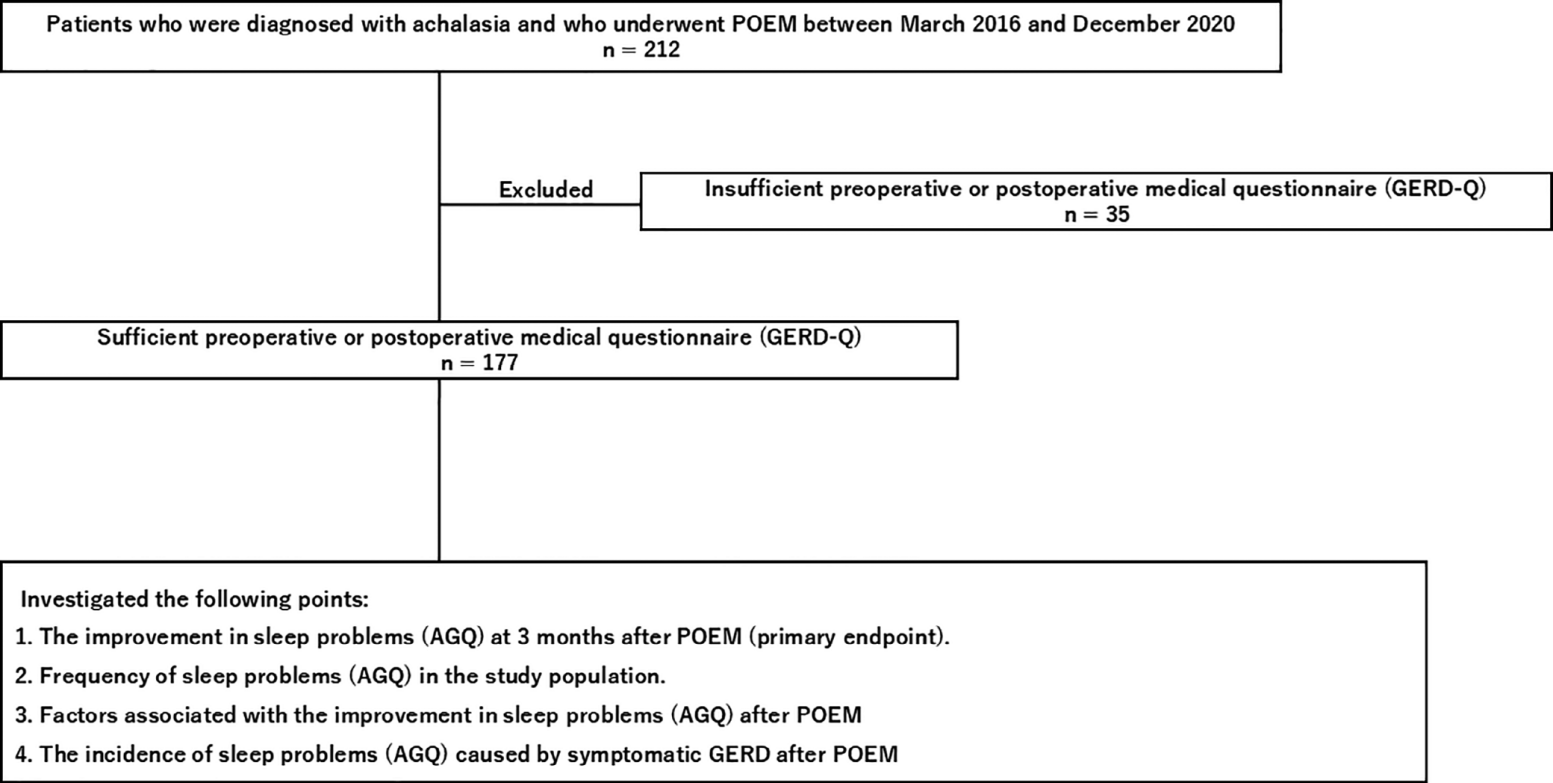
Study flowchart. A total of 212 patients were diagnosed with achalasia and underwent POEM between March 2016 and December 2020. However, because of insufficient preoperative and postoperative questionnaires, only 177 patients were included in the study. POEM, peroral endoscopic myotomy.

In this study, we retrospectively analyzed the data of consecutive patients who were diagnosed with achalasia and who underwent POEM at a single institution (Department of Gastroenterological Surgery, Fukuoka University Faculty of Medicine) between March 2016 and December 2020. Figure 1 shows the study flowchart. The primary endpoint was to examine the efficacy of POEM in improving sleep problems (AGQ) at 3 months after the POEM procedure. The secondary endpoints were to examine the frequency of sleep problems (AGQ) in patients with achalasia and to determine the factors associated with the improvement in sleep problems (AGQ) in these patients after POEM.

The diagnosis of achalasia was established through interviews, endoscopy, esophagography, high‐resolution manometry, and computed tomography. Interviews involved administering 1) the Eckardt symptom score assessment before and after POEM (Tables 1a); and 2) the GERD‐Q before and after POEM (Table 1b).^4^, ^5^

**TABLE 1a:** Eckardt symptom score.

	Symptom			
Score	Weight loss (kg)	Dysphagia	Retrosternal pain	Regurgitation
0	None	None	None	None
1	<5	Occasional	Occasional	Occasional
2	5–10	Daily	Daily	Daily
3	>10	Each meal	Each meal	Each meal

**TABLE 1b:** The GERD‐Q questionnaire respondents enter the frequency scores after reflecting on their symptoms over the previous week.

Question	Frequency score (points) for symptom			
	0 day	1 day	2–3 days	4–7 days
1. How often did you have a burning feeling behind your breastbone (heartburn)?	0	1	2	3
2. How often did you have stomach contents (liquid or food) moving upward to your throat or mouth (regurgitation)?	0	1	2	3
3. How often did you have pain in the centre of the upper stomach?	3	2	1	0
4. How often did you have nausea?	3	2	1	0
**5. How often did you have difficulty getting a good night's sleep because of your heartburn and/or regurgitation?**	0	1	2	3
6. How often did you take additional medication for your heartburn and/or regurgitation, other than what the physician told you to take? (such as Tums, Rolaids, Maalox?)	0	1	2	3

To assess the efficacy of POEM in improving sleep problems (AGQ) at 3 months postoperatively (primary endpoint), as well as determine the factors associated with the improvement in sleep problems (AGQ; secondary endpoint), one of the items from the GERD‐Q was used.^5^ Specifically, one question regarding sleep (Q5: “How often did you have difficulty getting a good night's sleep because of your heartburn and/or regurgitation?”) was analyzed. A response of “0 days” indicated “no sleep problem (AGQ),” while any other response indicated “sleep problem (AGQ).” The frequency of each symptom was investigated using the Eckardt symptom score and the GERD‐Q.^4^, ^5^ The F scale was also used as a questionnaire to evaluate post‐POEM gastroesophageal reflux disease (GERD).^6^ Symptomatic GERD was diagnosed with an F scale score of 8 or higher. Factors associated with the improvement in sleep problems (AGQ) were identified by comparing the pre‐ and post‐POEM scores for each symptom. Specifically, we analyzed the changes in each symptom before and after POEM. Cases where the post‐POEM scores were lower than the pre‐POEM scores were classified as ‘improved,’ while cases where the scores remained the same or increased were classified as ‘non‐improved.’ Statistical analysis was then performed based on these classifications. After POEM, the incidence of sleep problems (AGQ) caused by symptomatic GERD was considered by dividing patients with sleep problems (AGQ) into those with and without symptomatic GERD. Patients who did not complete the questionnaires before and after POEM were excluded from the analysis.

The study was conducted with the approval of the Ethics Committee of Fukuoka University (approval number: U19‐04‐002) and in accordance with the Declaration of Helsinki.

### POEM procedure and follow‐up

The POEM procedure and follow‐up after the POEM were consistent with previous reports.^7^, ^8^, ^9^, ^10^, ^11^ All procedures were performed or supervised by an expert who had conducted more than 100 POEM procedures. POEM was performed under general anesthesia with endotracheal intubation, and the patients were in the supine position. Carbon dioxide insufflation was used during endoscopy. An electrosurgical triangle tip knife with an integrated water jet function (KD‐645L; Olympus Corp.) was the preferred surgical device, whereas a FlushKnife BT (DK2620J; Fujifilm) or SB Knife Jr. (MD‐47703L; Olympus Corp.) was used when necessary. Posterior myotomy was the primary choice, and the myotomy length was determined based on the type of achalasia. The double‐scope method was routinely performed to confirm the LES incision.^9^, ^12^

The patients were prescribed acid‐suppressing medications for 1 month after POEM as part of the standard procedure. Patients who were already taking acid‐suppressing medications for any reason continued using prescribed acid‐suppressing 1 month after the POEM procedure. Three months later, a questionnaire to assess the Eckardt symptom score, GERD‐Q, and F scale was administered, and endoscopy and manometry were performed.^4^, ^5^, ^6^

### Statistical analysis

Categorical data are presented as numbers (%), while continuous data are presented as the mean ± standard deviation. To examine the factors associated with the improvement in sleep problems (AGQ), we performed univariate logistic regression analysis using sleep improvement as a response variable. The statistical analyses were performed using SAS software (version 9.4; SAS Institute, Cary, NC). A *p*‐value of < 0.05 was considered statistically significant. All data were analyzed at an independent facility (Inter Scientific Research Co., Ltd., Japan).

## RESULTS

### Patients’ background and perioperative characteristics

A total of 212 patients were diagnosed with achalasia and underwent POEM between March 2016 and December 2020. However, because of insufficient preoperative and postoperative questionnaires, only the data of 177 patients were analyzed in this study.

The background and perioperative characteristics of the patients are shown in Table 2. The average age of the patients was 52.6 ± 17.2 years (range 13–87 years), with a sex distribution of 87 males and 90 females. The average disease duration was 108.0 ± 131.3 months. In terms of the types of achalasia, 131 of 177 patients (74.0%) were classified as straight type, 38 of 177 (21.5%) as sigmoid type, and eight of 177 (4.5%) as advanced sigmoid type. In the Chicago classification, Type I accounted for 20 of 171 (11.7%), Type II accounted for 129 of 171 (75.4%), Type III accounted for 11 of 171 (6.4%), and “other” accounted for 11 of 171 (6.4%).^13^, ^14^ Six patients were unable to undergo manometry.

**TABLE 2:** Patients’ background and perioperative characteristics (*n* = 177).

Characteristic	Value
Age, years	52.6 ± 17.2
Sex, female/male ratio	90/87
Disease duration, months	108.0 ± 131.3
Type of achalasia	
Straight	131/177 (74.0)
Sigmoid	38/177 (21.5)
Advanced sigmoid	8/177 (4.5)
Chicago classification	
Type I	20/171 (11.7)
Type II	129/171 (75.4)
Type III	11/171 (6.4)
Others	11/171 (6.4)
Previous treatment	
Pneumatic dilation	31/177 (17.5)
Heller‐Dor operation	7/177 (3.9)
POEM	1/177 (0.6)
Length of myotomy during POEM, cm	
Esophageal	12.8 ± 5.0
Gastric	2.4 ± 1.9
IRP, mmHg	
Before POEM	33.6 ± 15.9
After POEM	15.2 ± 5.6
Eckardt symptom score	
Before POEM	5.9 ± 2.4
After POEM	1.1 ± 1.1

The data are presented as the mean ± standard deviation or *n* (%) unless otherwise specified.

Abbreviations: IRP, integrated relaxation pressure; POEM, peroral endoscopic myotomy.

Among the patients, 39 of 177 (22%) had a previous history of treatment, including balloon dilation in 31 of 177 patients (17.5%), Heller‐Dor operation in seven of 177 patients (3.9%), and prior POEM in 1 of 177 patients (0.6%). The length of the myotomy on the esophageal and gastric sides was 12.8 ± 5.0 and 2.4 ± 1.9 cm, respectively. The integrated relaxation pressure was 33.6 ± 15.9 mmHg before POEM and 15.2 ± 5.6 mmHg after POEM. The Eckardt symptom score was 5.9 ± 2.4 before surgery and 1.1 ± 1.1 after surgery.

### Symptoms associated with achalasia

**FIGURE 2:**
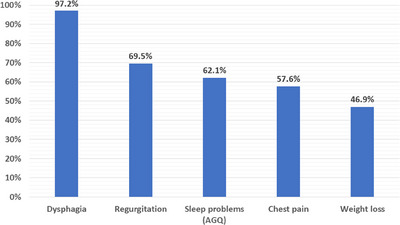
Frequency of symptoms associated with achalasia (*n* = 177). Among the symptoms of achalasia, sleep problem (AGQ) was the third most common.

In patients with preoperative achalasia, the following symptoms were observed (Figure 2): dysphagia (172 of 177 [97.2%]), regurgitation (123 of 177 [69.5%]), sleep problems (AGQ; 110 of 177 [62.1%]), chest pain (102 of 177 [57.6%]), and weight loss (83 of 177 [46.9%]).

### Improvement in sleep problems (AGQ) at 3 months after POEM (primary endpoint)

**FIGURE 3:**
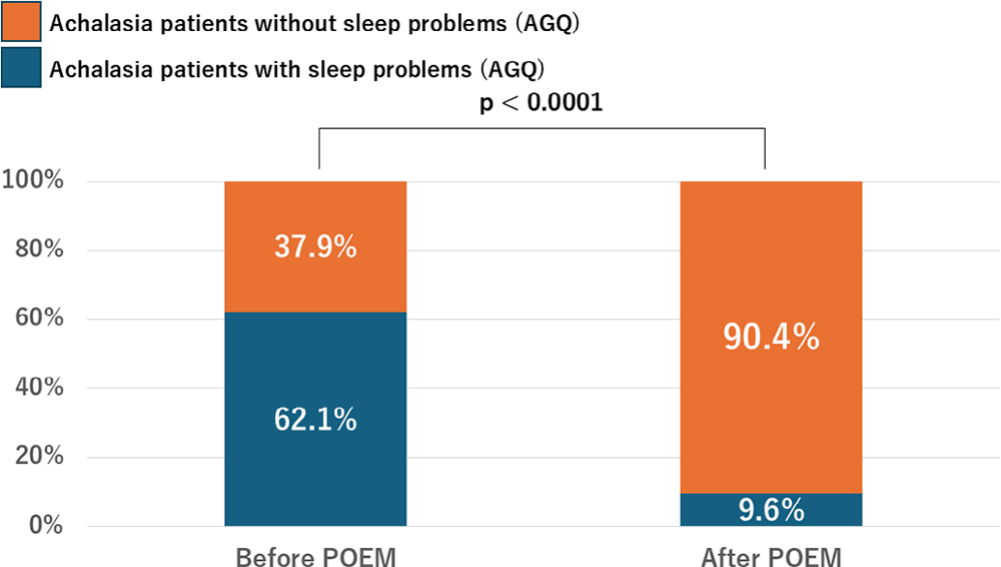
Effect of peroral endoscopic myotomy (POEM) on sleep problems (AGQ) at 3 months postoperatively (*n* = 177). Before POEM, 62.1% of patients experienced sleep problems (AGQ). After the POEM procedure, 9.6% of patients continued to have sleep problems (AGQ; *p* < 0.0001). POEM, peroral endoscopic myotomy.

Before POEM, 110 patients (62.1%) experienced sleep problems (AGQ). After POEM, 17 patients (9.6%) continued to have sleep problems (AGQ; *p* < 0.0001; Figure 3).

### Factors associated with the improvement in sleep problems (AGQ) after POEM

The univariate logistic regression analysis was performed on patients with preoperative sleep problems (AGQ), with the presence of postoperative sleep problems (AGQ) being concluded based on the improvement or non‐improvement in the scores for dysphagia, regurgitation, chest pain, and weight loss as the explanatory variables. The results revealed significant differences in dysphagia and regurgitation (*p* < 0.05; Table 3).

**TABLE 3:** Factors associated with the improvement in sleep problems (AGQ) after POEM.

	Change in Eckardt symptom score after POEM	Sleep problem (AGQ)		Logistic regression analysis		
		No	Yes	OR	95％ CI	*p*
Dysphagia	Non‐improved	69.2% (9)	30.8% (4)	Reference	1.400–23.332	0.0151*
	Improved	92.8% (90)	7.2% (7)	5.716		
Regurgitation	Non‐improved	73.3% (11)	26.7% (4)	Reference	1.151–18.155	0.0308*
	Improved	92.6% (88)	7.4% (7)	4.571		
Chest pain	Non‐improved	87.0% (40)	13.0% (6)	Reference	0.506–6.196	0.3717
	Improved	92.2% (59)	7.8% (5)	1.770		
Weight loss	Non‐improved	88.2% (30)	11.8% (4)	Reference	0.358–4.828	0.6805
	Improved	90.8% (69)	9.2% (7)	1.314		

The data are presented as *n* (%).Univariate logistic regression analysis with improvement in sleep problems (AGQ) as the response variable *p < 0.05. OR, odds ratio.

### The incidence of sleep problems (AGQ) caused by symptomatic GERD after POEM

**FIGURE 4:**
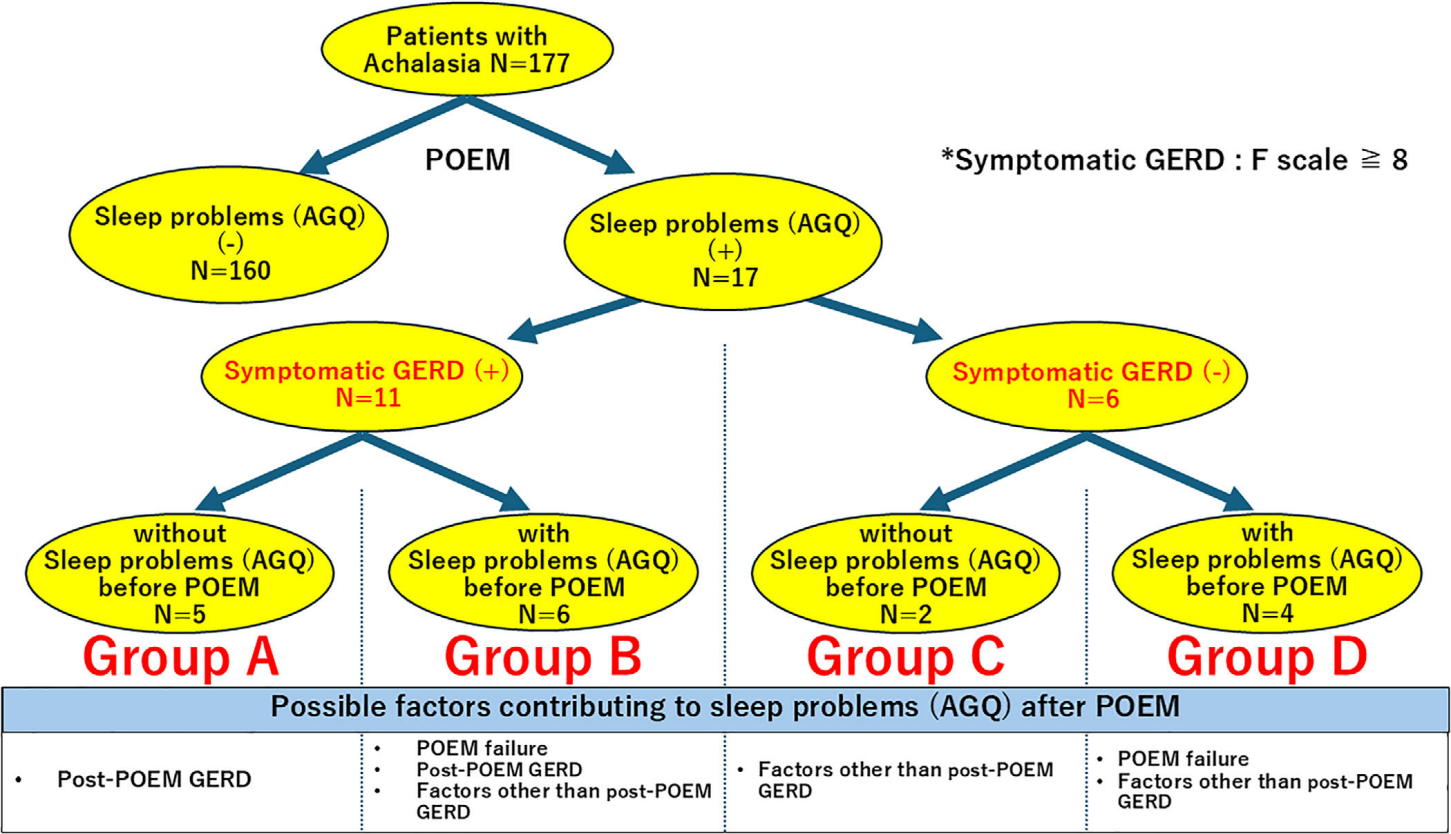
The incidence of sleep problems (AGQ) caused by symptomatic gastroesophageal reflux disease (GERD) after peroral endoscopic myotomy (POEM). Patients with sleep problems (AGQ) 3 months after POEM were categorized into Groups A–D based on the presence or absence of symptomatic post‐POEM GERD and pre‐POEM sleep problems (AGQ). Symptomatic GERD was diagnosed in patients with an F‐scale score of 8 or higher at 3 months after POEM. Possible factors contributing to sleep problems (AGQ) after POEM are indicated under each group. The occurrence of sleep problems (AGQ) related to symptomatic GERD after POEM was estimated to be 5–11 out of 177 patients (2.82%–6.21%), assuming that all patients in Group B had sleep problems (AGQ) due to symptomatic GERD. In this study, there were no cases of sleep problems (AGQ) caused by POEM failure.

Patients with sleep problems (AGQ) 3 months after POEM were classified into Groups A–D based on the presence or absence of symptomatic GERD after POEM and sleep problems (AGQ) before POEM (Figure 4). Symptomatic GERD was diagnosed in patients with an F‐scale score of 8 or higher at 3 months after POEM. Possible factors contributing to sleep problems (AGQ) after POEM are indicated under each group. The occurrence of sleep problems (AGQ) related to symptomatic GERD after POEM was estimated to be 5–11 out of 177 patients (2.82%–6.21%), assuming that all patients in Group B had sleep problems (AGQ) due to symptomatic GERD. In this study, there were no cases of sleep problems (AGQ) caused by POEM failure.

## DISCUSSION

Sleep is an important factor contributing to human health, and sleep problems are known to significantly reduce the quality of life of affected individuals.^15^ In the United States, 14.5% of adults have difficulty falling asleep.^16^ According to the Ministry of Health, Labor, and Welfare in Japan, it is estimated that approximately 30%–40% of the general adult population experiences some form of insomnia, with chronic insomnia affecting approximately 10% of adults.^17^

In the present study, a notably high proportion of patients with achalasia reported experiencing sleep problems (AGQ; *n* = 110 [62%], Figure 2). Moreover, the results showed that sleep problems (AGQ) improved significantly following the POEM procedure (from 62% to 9.6%, Figure 3), suggesting a causal relationship between achalasia and sleep problems (AGQ). Sleep problem (AGQ) was also the third most common symptom of achalasia. In patients with achalasia, sleep problems (AGQ) may be caused by 1) awakening during sleep due to dysphagia, regurgitation, and/or chest pain, 2) difficulty in falling asleep due to anxiety, or 3) brain excitation due to hormonal abnormalities caused by insufficient food intake (similar to those associated with dieting).^18^ Furthermore, sleep problems (AGQ) may arise from a combination of multiple factors rather than a single one. We investigated the factors associated with the improvement of sleep problems (AGQ). Our findings indicate that the improvement of dysphagia and regurgitation before POEM led to improvements in sleep problems (AGQ). These results highlight the importance of improving dysphagia and regurgitation with POEM to improve sleep problems (AGQ) in patients with achalasia. To effectively address dysphagia and regurgitation, it is crucial to address the inadequate LES relaxation and/or abnormal esophageal body contraction, both of which contribute to these symptoms.

Among the patients included in the present study, Chicago classification Type I and Type II were identified in 149 of 171 patients (87.1%). In all patients, we ensured complete incision of the LES using the double‐scope method during the POEM procedure. We believe that this approach played a significant role in the improvement of dysphagia and regurgitation, which consequently led to the amelioration of sleep problems (AGQ) associated with achalasia. In patients classified as Type III according to the Chicago classification, dysphagia and regurgitation are caused by abnormal esophageal body contraction in addition to inadequate LES relaxation. In patients classified as Type III, we believe that achieving an adequate myotomy length in the esophageal body, guided by endoscopy, barium esophagography, and manometry, along with the aforementioned double‐scope method, contributed similarly to the improvement in dysphagia, regurgitation, and even sleep problems (AGQ).

In Figure 4, patients with sleep problems (AGQ) 3 months after POEM were categorized into Groups A to D based on the presence or absence of symptomatic post‐POEM GERD and pre‐POEM sleep problems (AGQ). At 3 months post‐POEM, 17 patients were found to have sleep problems (AGQ). Of these 17 patients, 11 had symptomatic GERD, while six did not. Group A included five patients (five of 177 [2.82%]) who had no sleep problems (AGQ) before POEM but developed both sleep problems (AGQ) and symptomatic GERD afterward. It was considered that sleep problems (AGQ) in this group were caused by symptomatic GERD after POEM. Group B included six patients (six of 177 [3.39%]) who had sleep problems (AGQ) before POEM and had symptomatic GERD and sleep problems (AGQ) after POEM. It was considered that the sleep problems (AGQ) caused by achalasia were either not improved by POEM, or that although POEM improved the achalasia‐related sleep problems (AGQ), new sleep problems (AGQ) might have developed due to symptomatic GERD or other factors in this group. Group C included two patients (two of 177 [1.13%]) who had no sleep problems (AGQ) before POEM but had sleep problems (AGQ) without symptomatic GERD after POEM. Because symptomatic GERD was absent, sleep problems (AGQ) in this group after POEM were likely caused by factors other than GERD. Group D included four patients (four of 177 [2.26%]) who had sleep problems (AGQ) both before and after POEM but did not have symptomatic GERD. It was considered that the sleep problems (AGQ) caused by achalasia were either not improved by POEM, or that although POEM improved the achalasia‐related sleep problems (AGQ), new sleep problems (AGQ) might have developed due to factors other than symptomatic GERD. In this study, there were no cases of sleep problems (AGQ) caused by POEM failure. Based on these results, the occurrence of sleep problems (AGQ) related to symptomatic GERD after POEM is estimated to be 5–11 out of 177 patients (2.82%–6.21%), even if we assume that all patients in Group B had sleep problems (AGQ) due to symptomatic GERD. However, as mentioned later in the Limitations, this study did not consider important factors such as sleep disorders (e.g., sleep apnea or insomnia), the use of sleep medications, alcohol or smoking history, and specific sleep problems (e.g., difficulty falling asleep or early morning awakening). Therefore, I believe these figures might be regarded as reference values.

Currently, the Eckardt symptom score is widely used in POEM to evaluate the effectiveness of achalasia treatment and compare it with traditional treatment methods, such as Heller‐Dor surgery and balloon dilation.^19^, ^20^ However, the Eckardt symptom score does not include all important measures related to the quality of life of patients, such as sleep problems, as examined in this study. Additionally, weight loss, which is one of the items included in the Eckardt symptom score, does not occur after POEM, and chest pain cannot be distinguished from pain caused by GERD. Therefore, the appropriateness of the Eckardt symptom score in determining the effectiveness of POEM treatment is questionable. The primary goal of achalasia treatment is to improve the overall quality of life of affected individuals; therefore, it is hoped that the Eckardt symptom score can be further developed to incorporate quality‐of‐life assessments. In such cases, the evaluation of sleep problems, which was the focus of the present study, will be necessary.

This study has several limitations that should be noted. First, an overall assessment of sleep was not performed. In this study, “sleep problems (AGQ)” were evaluated only through the GERD‐Q, which is not specifically designed to assess sleep. GERD‐Q Q5 asks whether heartburn or reflux affects sleep. Furthermore, important factors such as sleep disorders (e.g., sleep apnea or insomnia), use of sleep medication, alcohol or smoking history, and specific sleep problems (e.g., difficulty falling asleep or early morning awakening) were not considered. Additionally, objective data indicating sleep such as electroencephalography during sleep, were not collected. Therefore, the content and causes of sleep problems (AGQ) were not evaluated through more objective methods beyond the questionnaire. Second, this was a single‐center retrospective study, which may limit the generalizability of the findings to other settings or populations. Third, the data collected were only short‐term, with follow‐up conducted 3 months after the POEM procedure. Longer‐term data would provide a more comprehensive understanding of the effects of the POEM procedure on sleep problems (AGQ) in patients with achalasia. Fourth, multivariate analysis was not performed in this study. Finally, we hope that future studies address these limitations with longer‐term follow‐up periods, specialized questionnaires for sleep problems, and objective data specific to sleep problems (such as electroencephalography), to provide a more robust understanding of the relationship between achalasia and sleep problems.

## CONCLUSION

Sleep problems assessed by GERD‐Q (AGQ) were observed in more than 60% of patients with achalasia in the present study, ranking as the third most common symptom. Improving dysphagia and regurgitation using the POEM procedure improved sleep problems (AGQ).

## CONFLICT OF INTEREST STATEMENT

None.

## ETHICS STATEMENT

The study protocol was approved by the Ethics Committee of Fukuoka University (approval number: U19‐04‐002). Informed consent was obtained through an opt‐out procedure. This study was not registered in a public registry, as it was not applicable. No animal studies were involved in this research.

## Data Availability

None.
